# The clinical effective of sodium/glucose co-transporter-2 inhibitors on admission frequency, duration and use of acute non-invasive ventilation in patients with chronic obstructive pulmonary disease: A single-centre 24-month retrospective observational study in a UK tertiary care centre

**DOI:** 10.1016/j.clinme.2025.100541

**Published:** 2025-12-03

**Authors:** Alan Kan, Joshua De Soyza, Opeyemi Kafi, Ranganatha Rao, Narasimha Murthy, Jayanth Bhat

**Affiliations:** University Hospital Coventry and Warwickshire, NHS Trust, Coventry, United Kingdom

**Keywords:** COPD exacerbation, SGLT2 inhibitors, Small airway disease, Hospital admissions, Length of stay

## Abstract

**Background:**

Chronic obstructive pulmonary disease (COPD) contributes significantly to global morbidity and mortality and high economic burdens to healthcare systems. Emerging evidence suggests that sodium/glucose co-transporter-2 inhibitors (SGLT2i), beyond their use in type 2 diabetes mellitus (T2DM) and heart failure (HF), offer multifaceted immunomodulatory and anti-inflammatory effects which may offer positive outcomes in patients with COPD.

**Objective:**

Investigate the impact of SGLT2i on inpatient outcomes – specifically, hospital length of stay (LOS), admission frequency, and use of acute non-invasive ventilation (NIV).

**Methods:**

We conducted a 24-month retrospective observational study of adults with spirometry-confirmed COPD admitted to University Hospitals Coventry and Warwickshire between April 2022 and April 2024. Patients receiving SGLT2i for ≥30 days prior to study start formed the treatment group. Multivariable Poisson logistic regression was used to analyse the association between SGLT2i use and key outcomes, adjusting for demographics, disease severity, comorbidities and concurrent therapies.

**Results:**

About 1,197 admissions of 627 patients met the inclusion criteria, with 32 patients (5%) prescribed SGLT2i. SGLT2i use was associated with a statistically significant 26% reduction in hospital LOS (95% CI 0.62–0.87; *p* ≤ 0.001), independent of co-existing HF or T2DM. However, there was no significant reduction in admission frequency (IRR 0.84, 95% CI: 0.62–1.09, *p* = 0.212) or acute NIV use (OR 2.62, 95% CI: 0.35–13.26, *p* = 0.277) among SGLT2i users.

**Conclusions:**

SGLT2i therapy in COPD patients was associated with a significantly reduced hospital length of stay during exacerbations, regardless of underlying heart failure or diabetes status. However, no differences were observed in admission frequency or acute NIV utilisation. These findings support the hypothesis of a beneficial anti-inflammatory or cardiopulmonary effect of SGLT2i in COPD and warrant further investigation through prospective trials to explore their therapeutic role in this population.

## Highlights

• Sodium glucose co-transporters-2 (SGLT2) inhibitors are associated with a reduction in length of inpatient admission.
• Increasing age and increased severity of FEV_1_ is associated with increased length of inpatient admission.
• SGLT2 inhibitors use did not correlate with a reduction in acute NIV or frequency of admissions.

## Graphical abstract

**Figure:**
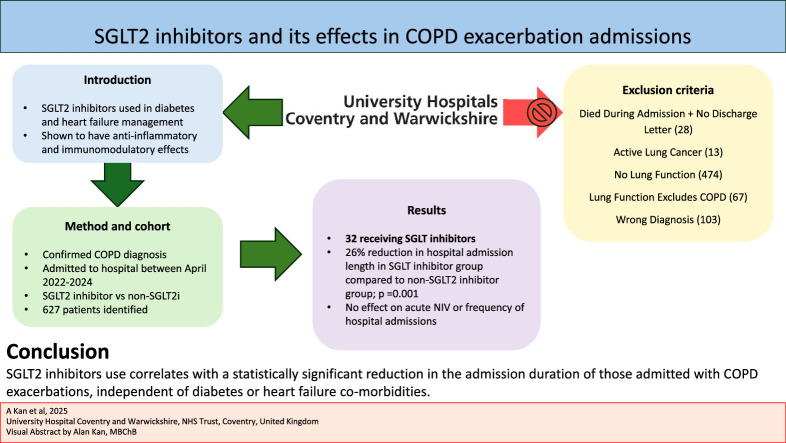

## Introduction

Chronic obstructive pulmonary disease (COPD) is a progressive disease that is estimated to be responsible for 5% of annual global deaths, and is the fourth leading cause of death worldwide.^1^ In the UK, the economic burden on the NHS on prevention and treatment measures in COPD is estimated at £3 billion annually,^2^ with estimated deaths in England projected to rise to 129,400 in 2030.^3^ Current preventative strategies focus on early detection, smoking cessation, optimised inhaler therapies and pulmonary rehabilitation.^4^

Airway inflammation in COPD is secondary to both innate and adaptive immunity, primarily affecting the small airways and leading to emphysema and chronic bronchitis.

The inflammation persists despite smoking cessation and is marked by progressive small airway obstruction driven by immune cell infiltration and inflammatory mucous accumulation.^5^ Inflammatory mediators and cytokines such as chemokines, interleukins (IL-6, IL-1B), tumour necrosis factor alphas (TNF-α) and acute phase proteins (CRP) play a pivotal role,^6^ with greater circulating inflammatory mediators being associated with a greater degree of systemic inflammation.^7^ Patients with COPD have a five times higher risk of cardiovascular disease and cardiovascular-related deaths, with a direct correlation between COPD severity and severity of cardiovascular disease.^8^ This is characterised by the development of atherosclerosis and coronary artery disease though the counter-play of pro-inflammatory mediators and cells involved in vessel endothelial damage and dysfunction.^9^

Sodium/glucose co-transporter-2 inhibitors (SGLT2i) are established therapeutics in type 2 diabetes and prevent resorption of glucose from the renal proximal tubule system, thus acting as a powerful diuretic.^10^ SGLT2i also have multifaceted, immunomodulatory and anti-inflammatory effects,^11^ with recent experimental studies demonstrating a reduction in pro-inflammatory cytokines such as TNF-α, IL-6 and IL-1.^12^ It is this mechanism that is hypothesised to lead to the cardioprotective properties of SGLT2i, by reducing oxidative stress in cardiomyocytes, reducing cardiac hypertrophy and improving blood pressure control by reducing cardiac pre- and afterload,^13^, ^14^, ^15^ and the wider implications in other cardiac conditions such as valvular and coronary heart disease, cardiomyopathies and cerebrovascular disease are just starting to be identified.^16^ The use of SGLT2i in patients with COPD with diabetes has also been linked to significantly lower risks of acute COPD exacerbations, hospitalisation rates and mortality in admissions secondary to COPD exacerbations compared to those not receiving SGLT2i, although the mechanism is not fully understood.^17^, ^18^, ^19^

We, therefore, aim to investigate to see what effect SGLT2i have on our COPD population with specific factors such as a) length of stay and b) frequency of admissions in patients compared to other anti-glycaemic agents (or none) within our population subset at University Hospitals Coventry and Warwickshire NHS Trust, UK.

## Aim

Our aim was to conduct a single-centre retrospective study into SGLT2i and their effects in patients with known diagnoses of COPD who were admitted into University Hospitals Coventry and Warwickshire between April 2022 and April 2024 with infective and non-infective exacerbations. Specifically, we wanted to analyse how many of these patients were receiving SGLT2i at the time of their admission, and whether the prescription of SGLT2i had any advantages, primarily focusing on a) length of stay and b) frequency of admissions.

## Methods

### Data source

Patients were identified from clinical codes within digital discharge summaries. All those with a clinical code of COPD exacerbation were included. Data were collected from these patients’ electronic records. Collected data included patient demographics, primary diagnosis during admission, comorbidities, medications aligned to their respiratory disease, and pulmonary function results.

### Population

The study population was divided into two groups, with the control group reserved for patients receiving either normal anti-glycaemic or none, and the study group reserved for those taking SGLT2i either alone or in combination with other anti-glycaemic agents.

### Inclusion and exclusion and measurable outcomes

The study focused on analysing the frequency of admissions, length of hospital stays and use of non-invasive ventilation during admissions for each patient group.

Patients included were those admitted between 1 April 2022 and 30 April 2024, with a confirmed COPD exacerbation diagnosis on discharge, who received both steroid and antimicrobial therapy during admission, had a formal COPD diagnosis via post-bronchodilator spirometry or has been followed up in an outpatient COPD clinic regardless of smoking history, and were either not receiving the specified anti-glycaemic agents (control group) or had been for at least 30 days prior to 1 April 2022 (study group).

**Fig. 1:**
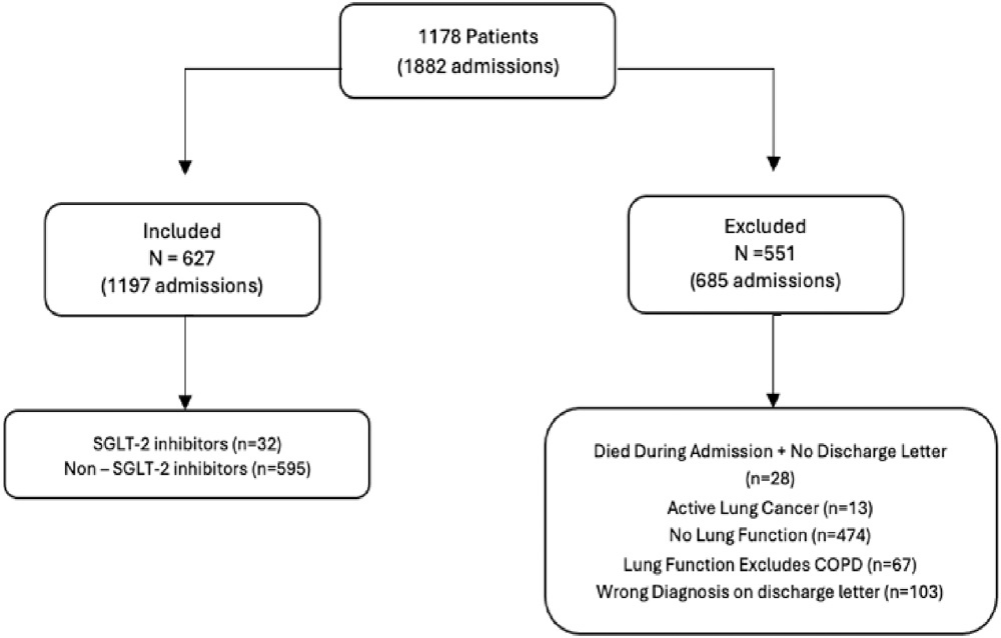
Flow diagram demonstrating the number of patients included (627) and excluded (551) in the study. The exclusion criteria are shown with the corresponding number of admissions (685) that were excluded in each category.

The exclusion criteria included absence of spirometric confirmation of obstructive airway disease, use of anti-glycaemic agents for less than 30 days during the study period, and patients with liver failure or on regular dialysis for end-stage renal failure. Patients newly diagnosed with congestive heart failure, ischaemic heart disease, cerebrovascular events, or lung cancer within 3 months of the study start were also excluded to minimise latent disease effects. Additionally, patients without a discharge letter confirming COPD exacerbation or those undergoing active chemotherapy or radiotherapy (excluding hormonal therapy) during the study period were excluded (Fig. 1). About ‘≥30 days’ was chosen to maximise the time to therapeutic effect by this class of drugs without restricting the number of patients identified who would qualify within the 24-month period. This was also chosen as few analyses have studied the time to benefit window for SGLT2i in type-2 diabetes and heart failure. Chen *et al*^20^ showed an observed statistical significance in reducing deterioration in heart failure or the risk of cardiac death at 26 days, whilst Vaduganathan *et al*^21^ showed statistically significant reductions in cardiovascular death or deterioration in heart failure after 16 days (in an analysis into the DELIVER trials). A smaller study into continuous glucose monitoring in type 2 diabetes and SGLT2i demonstrated a reduction in serum glucose after 2 h of ingestion, with sustained improvement in serum glucose levels variation 1 week after initiation.^22^

### Statistical methods

Demographics and use of NIV between the two groups were compared using standard statistical tests. Using R version 4.5,^23^ length of stay (LOS) was modelled using Poisson regression with a log link, as the outcome represented count data (days). Admission frequency (count of admissions) was analysed using a similar Poisson regression. NIV use (yes/no) was analysed using binary logistic regression. Multivariate models were used if initial univariate analysis showed significant results. Covariates included were age, sex, FEV_1%_ predicted, smoking status (ever vs never), presence of congestive cardiac failure, presence of type 2 diabetes mellitus, and co-prescriptions of inhaled corticosteroids, long-term macrolides and GLP-1 agonists. These variables were selected as they are known determinants in COPD exacerbations and length of stay, with the specific comorbidities chosen due to the use of SGLT2i in these disease populations.^24^ Model assumptions were checked for collinearity and homogeneity of variance. The association of GLP-1 agonists with median length of stay was assessed using simple statistical tests due to small numbers in the GLP-1 agonist group.

## Results

### Study population and inclusion criteria

Patients were identified from clinical codes within digital discharge summaries. All those with a clinical code of COPD exacerbation were included. Data were collected from these patients’ electronic records. Collected data included patient demographics, primary diagnosis during admission, comorbidities, medications aligned to their respiratory disease, and pulmonary function results.

### Admission characteristics

Most patients were admitted only once (363, 58%), while the median length of an admission was 4 days (63, 10%) with most admissions being 2 days in duration (87, 14%).

In both SGLT2i and non-SGLT2i cohorts, more ex-smokers than current smokers (317, 61% vs 245, 39%, respectively) were identified, with more patients (14, 44%) diagnosed with moderate COPD as per the GOLD criteria^25^ in the SGLT2i cohort compared with more with severe disease in the non-SGLT2i cohort (250, 42%) (Table 1).

**Table 1:** Demographic characteristics by SGLT2i use.

Variable	SGLT2i: Yes (*n* = 32)	SGLT2i: No (*n* = 595)	*p*-value
Mean age (years)^⁎^	73	72	0.5
Sex^⁎⁎⁎^			0.033
Female	10 (31%)	301 (51%)	
Male	22 (69%)	294 (49%)	
Smoking status^§^			0.5
Current	15 (47%)	230 (39%)	
Ex	17 (53%)	360 (61%)	
Never	0 (0%)	2 (0.3%)	
Pack year history^⁎⁎^	45 (25)	53 (33)	0.3
GOLD COPD severity^§^			0.5
Mild	1 (3.1%)	26 (4.4%)	
Moderate	14 (44%)	192 (32%)	
Severe	13 (41%)	250 (42%)	
Very severe	4 (13%)	126 (21%)	
Heart failure^⁎⁎⁎^	23 (72%)	133 (22%)	<0.001
Type 2 diabetes^⁎⁎⁎^	17 (53%)	98 (16%)	<0.001
GLP-1 agonist prescription^§^	2 (6.3%)	2 (0.3%)	0.014
Long-term macrolide prescription^§^	3 (9.4%)	58 (9.7%)	>0.9
Inhaled corticosteroid prescription^⁎⁎⁎^	28 (88%)	500 (84%)	0.6

SGLT2i, SGLT2-inhibitor; GOLD, Global Initiative for Chronic Obstructive Lung Disease; NIV, non-invasive ventilation.

1. Mean (SD) (^⁎^); *n* (%).

2. Wilcoxon rank sum test (^⁎⁎^); Pearson’s chi-squared test (^⁎⁎⁎^); Fisher’s exact test (^§^).

### Comorbidities and SGLT2 inhibitor prescription

We identified 32 (5%) patients receiving SGLT2i during our study period (male, *n* = 22; female, *n* = 10; dapagliflozin, *n* = 26; empagliflozin, *n* = 6) and four (0.6%) receiving GLP-1 agonists (dulaglutide, *n* = 3; liraglutide, *n* = 1).

There was a greater proportion of patients receiving SGLT2i with a diagnosis of either heart failure (23, 72%) or diabetes (17, 53%): an expected result due to the current licensing of SGLT2i in the management of both these conditions.

Of all 627 patients, 73 patients had an isolated diagnosis of heart failure, of these six (8%) were receiving SGLT2i. About 54 patients had an isolated diagnosis of T2DM, five (9%) receiving SGLT2i. About 83 had an isolated diagnosis of ischaemic heart disease, of which only one (1%) patient was receiving an SGLT2i.

A study of the combinational diagnoses of interest revealed 39 patients (6%) had both heart failure and ischaemic heart disease (seven patients receiving SGLT-2 inhibitors), 26 (4%) had heart failure and diabetes (six receiving SGLT-2 inhibitors), 17 (2%) with ischaemic heart disease and diabetes (two receiving SGLT-2 inhibitors) and 18 (3%) with a combination of all three comorbidities (one receiving SGLT-2 inhibitors). About 32 (5%) patients required acute non-invasive ventilation during their admission, with two of these patients being prescribed an SGLT-2 inhibitor (Table 2).

**Table 2:** Clinical outcomes by SGLT2 inhibitor prescription.

Outcome	SGLT2i: Yes (*n* = 32)	SGLT2i: No (*n* = 595)
Median length of stay (days, per patient)	4.7 (2.7)	6.1 (6.0)
Admission count	1.72 (1.11)	1.92 (1.67)
Inpatient NIV use	2 (6.3%)	32 (5.4%)

SGLT2i, SGLT2-inhibitor; NIV, non-invasive ventilation.

### Admissions duration, admission rates, and acute NIV

**Fig. 2:**
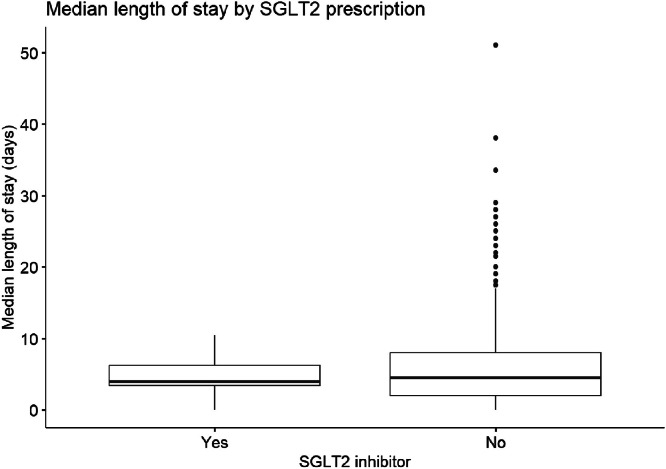
Boxplot showing median length of stay by SGLT2-inhibitor prescription. *n* = 627, *p* = 0.001.

In regression modelling, SGLT2i were associated with a 26% reduction in inpatient admission duration (CI 0.62–0.88, *p* = 0.001), accounting for age, sex, FEV_1_ and concurrent diagnosis of congestive heart failure and type 2 diabetes (Fig. 2; Table 3).

**Table 3:** Multivariate Poisson logistic regression model output of median length of stay.

Associations	Median length of stay (days)		
	Incidence rate ratios	CI	*p*
SGLT2-inhibitor prescription	0.74	0.62–0.88	0.001
Age (years)	1.02	1.02–1.02	<0.001
Sex (male vs female)	1.06	0.99–1.13	0.104
FEV_1%_ predicted	0.99	0.99–0.99	<0.001
Congestive cardiac failure	1.12	1.04–1.21	0.002
Type 2 diabetes mellitus	0.92	0.85–1.01	0.068
Long-term macrolide use	1.14	1.02–1.26	0.016

Observations	625		
R^2^ Nagelkerke	0.274		

SGLT2i, sodium/glucose co-transporter-2 inhibitors; CCF, congestive cardiac failure; T2DM, type 2 diabetes mellitus.

Our study showed there was no reduction in admission rates over the 24-month period in the SGLT2i cohort (IRR 0.84, CI: 0.62–1.09; *p* = 0.212) when compared to non-SGLT2i cohort. There was also no difference in utilisation of acute NIV between both groups on univariate analysis (OR 2.62, CI 0.35–13.26, *p* = 0.277).

Only four patients were identified as having been prescribed a GLP-1 agonist within our study population. There was no statistical significance in length of stay reduction in simple statistical tests between those prescribed GLP-1 agonists and those not (mean 4.4 vs 6.0 days, *p* = 0.932), possibly owing to the small group size.

## Discussion

SGLT2i are currently not licensed to be used as an adjunct therapy in COPD, but they are extensively prescribed in the management of type 2 diabetes, and demonstrated to reduce mortality risk and hospitalisations in congestive heart failure with reduced ejection fraction.^26^ We have shown in this 2-year retrospective study that the prescription of SGLT2i in our COPD patients was associated with a reduction in length of stay by 26% compared to the non-SGLT2i group, independently of heart failure or diabetes. There was, however, no statistical difference in the admission rates between the two groups.

Covariates largely had expected effects on median length of stay: those who were older had 2% increased length of stay with each additional year of age; those with poorer FEV_1%_ predicted values had 1% longer stays with each 1% deterioration; those with heart failure had 12% longer stays, possibly due to poorer exercise tolerance and therefore longer recovery times; and those with long-term macrolide usage had 14% longer stays. Direction of association is less intuitive in this last case, though those on long-term macrolide prescriptions will have had higher frequency of exacerbations, which correlates with length of hospital stay.^27^

This study supports the growing recent literature in the positive associations of use of SGLT2i in COPD exacerbations, from authors such as Gupta *et al*^29^, Shanmugavel *et al*^28^ and others. However, our study did not demonstrate any favourable outcomes between SGLT-2 inhibitor use and admission frequency or acute NIV, which differs from other studies.^19^^,^^28^ This could be due to the short study period evaluated, or low numbers of patients who were prescribed SGLT2i within our study. However, the work of other groups has shown a correlation in the use of SGLT2i and reduction in exacerbations requiring hospitalisation,^28^^,^^29^^,^^17^ and with reduced mortality.^19^ Although the mechanisms by which SGLT2i lead to these outcomes is still poorly understood, there may be an association between SGLT2i ability to reduce pro-inflammatory cytokines such as TNF-α, IL-6 and IL-1 and a reduction in airway inflammation severity.^11^^,^^12^

### Limitations

A limitation to this study was the omission of data from the outpatient cohort, such as those patients who are known to the COPD team in the community but were not admitted to UHCW during the 2022–2024 period. Some selection bias may therefore have been present, as we had a limited study period and may have missed patients who are only admitted very infrequently, but these likely constitute a minority and this does not significantly impact the strength of the data. One further limitation is the observational nature of the analysis presented here, as we cannot comment on the direction of association, ie whether SGLT2i caused a reduction in length of stay, or whether other factors are involved. For example, those who have been escalated to SGLT2i use for CCF or T2DM are likely to be those most engaged with healthcare in general, which may lead to reduced admission lengths by way of greater engagement with medical, nursing and physiotherapy treatment during their stay. Another limitation of the data was the inability to correct for BMI, socioeconomic status or vaccination status. Since both flu vaccination and underweight BMI have been proven to impact length of stay,^30^^,^^31^ this may conceivably have had a small impact on our data. A key limitation to highlight within this study is the small sample size, with only 32 patients identified receiving SGLT2i, and a smaller sample number receiving acute NIV. There is a risk of overinterpretation of the results, with an argument that the findings could be resultant of event randomisation rather than true events. This concern has to be acknowledged; therefore, we would advocate large-scale, multi-centre studies in SGLT2i in COPD to enable stronger statistical analysis on this topic.

We were robust in our approach in including only those with spirometrically confirmed COPD and excluding or attempting to correct those with other reasons for increased length of stay, as demonstrated by the association of long-term macrolide use on length of stay in the regression models, overall resulting in a relatively strong goodness-of-fit in the median length of stay model presented. Other factors not included in the model which may also influence length of stay include social factors such as availability of packages of care or financial and family support. In future research, such factors could be addressed by measuring length of stay from time admitted to time deemed ‘medically fit for discharge’.

There is acknowledgement that there is a discrepancy in the total population number of patients studied and the total number of patients’ smoking status that were recorded in Table 2 and the presence of two ‘never’ smokers. Three patients did not have a documented smoking history within their clinic letters, records from their general practitioner (GP) or discharge letters. It is more challenging to explain the presence of the two ‘never’ smokers within the study population; however, it can be postulated that their lung disease could be secondary to non-tobacco-related environmental risk factors aligned more with poor socio-economic status, infectious disease or occupational exposure to certain pollutants that would increase the risk of developing COPD.^32^

## Conclusion

As a retrospective observational study, our work adds to the growing body of literature that demonstrates improved outcomes in patients with COPD on an established treatment regimen of SGLT2i. Specifically, we have demonstrated that SGLT2i are associated with a significant reduction in the length of stay in COPD exacerbations, independent of heart failure or type 2 diabetes diagnoses. More substantially powered studies are required to better assess for any association with NIV or ICU intervention. This may lead to the development of future RCTs with aims to evaluate the potential role of SGLT2i in COPD patients without type 2 diabetes or congestive heart failure.

## CRediT authorship contribution statement

**Alan Kan:** Writing – review & editing, Writing – original draft, Supervision, Project administration, Methodology, Investigation, Formal analysis, Data curation, Conceptualization. **Joshua De Soyza:** Writing – review & editing, Writing – original draft, Methodology, Formal analysis. **Opeyemi Kafi:** Writing – review & editing, Data curation. **Ranganatha Rao:** Writing – review & editing, Supervision, Conceptualization. **Narasimha Murthy:** Writing – review & editing, Supervision, Conceptualization. **Jayanth Bhat:** Writing – review & editing, Supervision, Conceptualization.

## Declaration of competing interest

The authors declare that they have no known competing financial interests or personal relationships that could have appeared to influence the work reported in this paper.
